# A rare diaphragm in the common carotid artery: A first case report and literature review

**DOI:** 10.1097/MD.0000000000007331

**Published:** 2017-07-14

**Authors:** Ying Wang, Jian-chu Li, Ke Lv, Nai-li Wang, Gao-wa Sharen, Li Tan

**Affiliations:** a Department of Ultrasound, Chinese Academy of Medical Sciences & Peking Union Medical College Hospital; b Department of Human Anatomy, Histology and Embryology, Chinese Academy of Medical Sciences; c Department of Health Management, Peking Union Medical College Hospital, Beijing, China.

**Keywords:** CCA, diaphragm, variant

## Abstract

**Rationale:**

We present a case of common carotid artery (CCA) diaphragm. To our knowledge, this is the first reported case in the imaging literature.

**Patient concerns:**

A 33-year-old woman presented herself to an annual health examination without any report of ill health.

**Diagnoses:**

A stenosis with aneurysm dilation on the proximal part of the right CCA was revealed on ultrasound and CTA, by which a diaphragm-like structure on the stenosis lesion was clearly identified. An anatomic variant of artery was first considered.

**Interventions:**

The patient was followed up closely.

**Outcomes:**

There was no progression of her CCA stonosis 1 year later.

**Lessons:**

This unusual and previously unreported case is presented to alert vascular sonographers and radiologists to its existence.

## 1 Introduction

A diaphragm in the carotid artery is very rare. There were no case reports of common carotid artery (CCA) diaphragm reported and there were only 2 cases of internal carotid artery (ICA) diaphragm discovered by ultrasound reported in the literature. Rare forms of atherosclerosis and fibromuscular dysplasia (FMD) were considered separately. This article presents a previously undocumented CCA diaphragm revealed on cervical duplex ultrasound scanning and CTA and an anatomic variant of artery was first considered.

## 2 Case report

**Figure 1:**
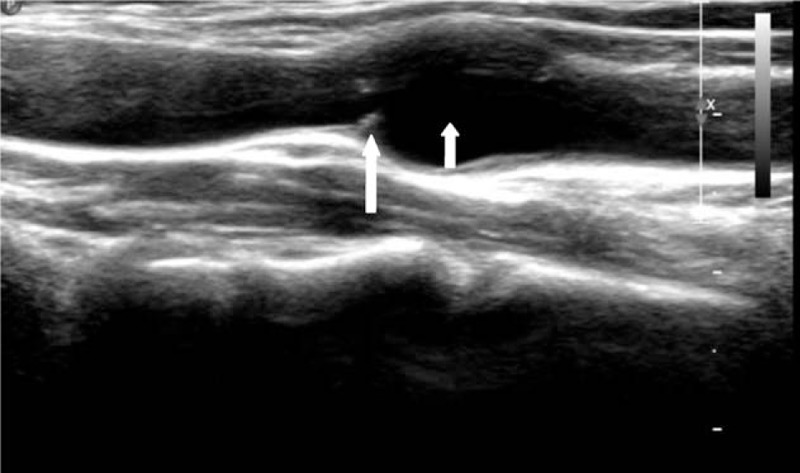
Color Doppler ultrasound scan showed an obviously localized dilation (short arrow) with a thin diaphragm (long arrow) with a small central lumen on the distal part of CCA. CCA = common carotid artery.

**Figure 2:**
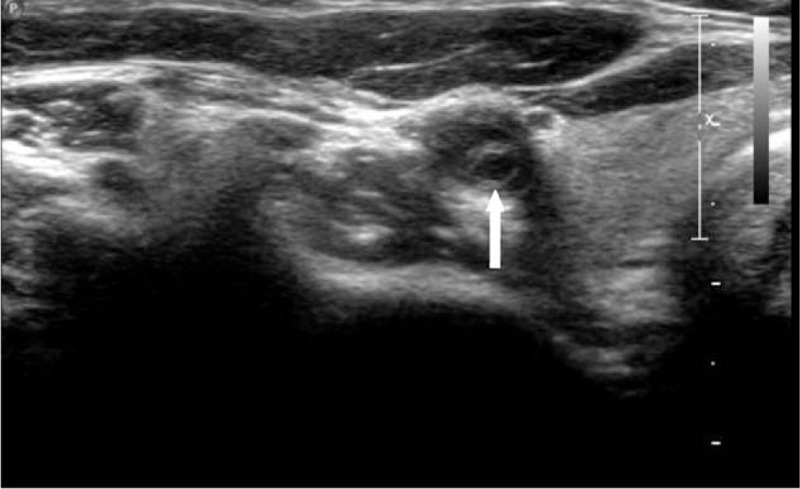
Transverse section of color Doppler ultrasound scan demonstrated the typical “fish mouth” (arrow) shape of diaphgram.

**Figure 3:**
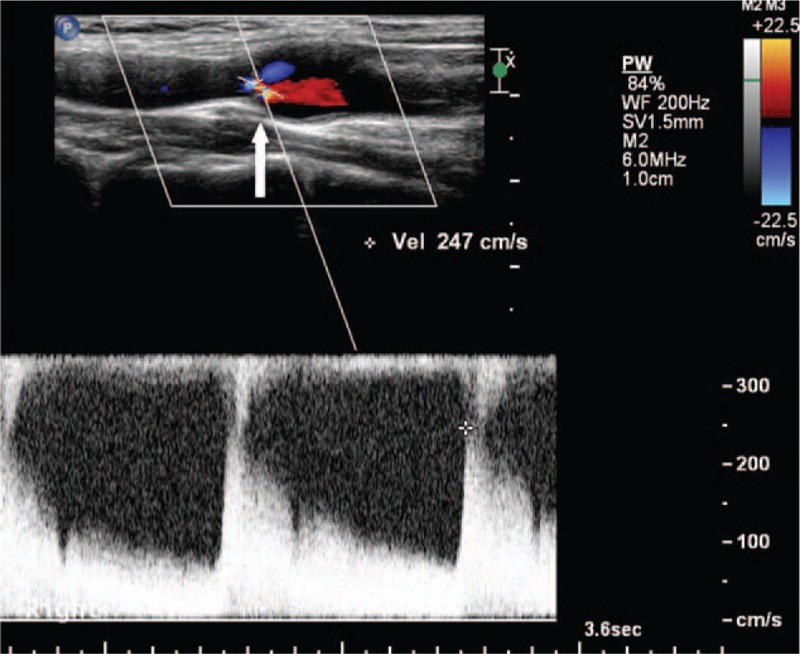
Color Doppler flow imaging (CDFI) showed turbulent color jet through this lumen of high velocity blood flow of 247 cm/s (arrow). CDFI = Color Doppler flow imaging.

**Figure 4:**
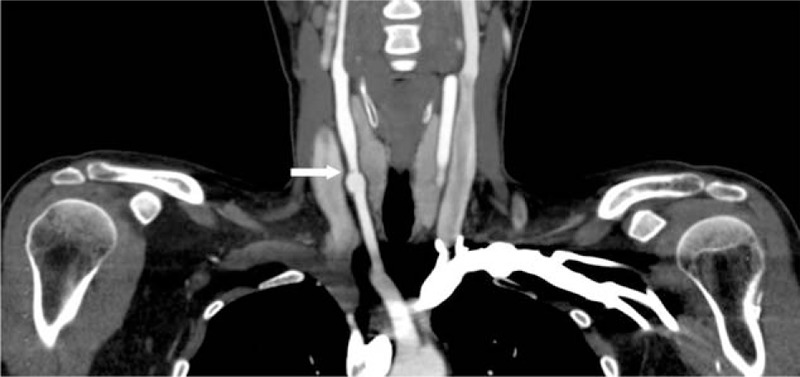
CTA showed a transversed, low density strip (arrow), causing luman stenosis, with proximal part aneurysmal enlargement on lower part of right CCA. CCA = common carotid artery, CTA = computed tomography angiography.

A 33-year-old woman presented herself an annual health examination, a carotid bruit was heard on the right CCA, whereas no other cardiovascular or neurological abnormality was demonstrated on physical examination. Her past medical history was noncontributory, with no hypertension, diabetes, or atherosclerosis. On laboratory examination, complete blood count, inflammatory markers, and blood lipid profiles were all within normal ranges. Then, she was referred for a carotid color Doppler ultrasound scan for further evaluation, founding an obviously localized dilation of the right CCA with an intact vascular wall structure, 1.3 cm in length, 1.1 cm in width, and 1.0 cm in height, located in the lower position of the artery (Fig. 1). Further observation on the longitudinal section showed there was a thin diaphragm with a small central lumen just on the distal part of aneurysm, and on transverse section, the diaphragm demonstrates the typical “fish mouth” shape (Fig. 2). The turbulent color jet with a high velocity blood flow of 247 cm/s through this lumen suggested that there was a tight stenosis (Fig. 3). For further evaluation, a carotid CTA scan was performed, showing a transversed, low density strip on lower part on the right CCA, causing lumen stenosis, with proximal part aneurismal enlargement, in consistent with what ultrasound scan found (Fig. 4). One-year follow-up with carotid color Doppler ultrasound scan performed by the same radiologist showed almost the same assessment result of the CCA stonosis. We presented the ultrasound and radiologic findings of this rare anatomic variant, a rare diaphragm was found in the right CCA. In addition, we conducted a review of the current literature, summarizing the anatomic, embryologic, and imaging implication.

## 3 Discussion

We searched the MEDLINE/PubMed/Embase databases using combinations of the keywords diaphragm, carotid web, carotid stenosis, and carotid artery for articles published since 1980. There were no case report of CCA diaphragm reported and there were only 2 cases of internal carotid artery (ICA) diaphragm discovered by ultrasound reported in the literature. In 2004, Raman reported a 70-year-old male suffered episodes of transient loss of vision in the left eye with a thin diaphragm across the carotid bulb in left ICA, a high velocity blood flow through this lumen suggested a greater than 70% stenosis.^[1]^ The patient underwent carotid endarterectomy and histology confirmed that the ICA athermanous plaque contained a thin diaphragm with a 1 mm central hole, which was composed of dense fibrous tissue while the central lumen was lined by endothelium. As the patient was symptomatic only at a late stage, it is thought that the fibrous diaphragm represents an unusual form of atherosclerosis rather than an anatomical variant. A recurrent diaphragm-like band of the left ICA as the cause of greater than 70% stenosis under duplex ultrasound was again reported in 2015 by Winnie who described a 76-year-old male suffered a syncopal episode upon waking up.^[2]^ A rare form of fibromuscular dysplasia (FMD) was considered by the author while no surgery was performed. In both cases, the carotid artery diaphragms were reported in old men with episodic symptoms and were both located in the left ICA

However, in our case, given the consideration of different onset age, clinical symptoms, and artery lesion location, we consider it is quite different from the former 2 cases. In our case, an asymptomatic 33-year-old woman incidentally found aneurismal enlargement with an intact vessel wall structure of CCA by ultrasound; further observation found a thin diaphragm with a small central lumen on the distal part of aneurysm, a finding supported by latest CTA investigation. The patient had no predisposing medical conditions such as hypertension, diabetes mellitus, atherosclerosis, or vasculitis. And common laboratory examinations were all in the normal range. In consideration of above, atherosclerosis disease and vasculitis were excluded. For such a young and asymptomatic woman, an anatomic variant of artery was first considered.

In normal carotid artery anatomy, the left and right CCAs follow the same course up through the neck. The vessel caliber remains constant because there is no side branches. The average vessel caliber was documented 8 mm by measurement of ultrasound and angiography.^[3,4]^ At the carotid bifurcation position, there is fusiform dilatation called carotid bulbar sinus. The embryology of carotid arteries system shows that it develops as a result of regression and combination of multiple arterial segments. Between days 21 to 32, 6 pairs of aortic arches develop and they connect the aortic sac to the dorsal aortae. Regression of the first 2 pair arches and part of the dorsal aorta forming a single conduit connecting the aortic sac to the cranial region,^[5]^ CCA and ICA system were formed by connection of aortic sac, third aortic arch, and dorsal aorta.^[6]^ The fourth arch arteries contribute to the asymmetry of the carotid origins, aoratic arch on the left and right subclavian artery on the right. The sixth arches form the proximal parts of the pulmonary arteries. At approximately 40 days, rapid descent of the heart causing the migration of the origin of the external carotid along the third arch determines the site of the carotid bifurcation.^[7]^ As a result of persistence, regression and migration of these arterial segments, especially the third and fourth arches, the carotid arterial system develops. There are no previous reports of CCA diaphragms, with all other CCA variations being anomalous carotid bifurcations.^[8,9]^ Except for them, there are few cases of absence of CCA reported worldwide.^[10–12]^ These anomalies are typically asymptomatic and usually discovered as an incidental finding. As reported, the absence of the CCA has no side or sex preferences and can occur bilaterally.^[11,12]^ One hypotheses accounting for the rare anatomy is that complete regression of the third bronchial arch and persistence of the ductus caroticus,^[13]^ which could share a common embryology process with our case. For our case, we hypotheses that may be CCA diaphragm is a one variant form of CCA absence, which means the regression of the third bronchial arch was not complete, forming a diaphragm like in our case rather than artery absence.

In addition, the aneurysm enlargement formation was considered to be associated with distal stenosis caused by the diaphragm, causing increasing localized blood vessel resistance, blood hemodynamic disturbance, and high pressure on the vessel wall on the proximal part of the diaphragm. Furthermore, we hypotheses that vessel enlargement of distal part could also be formed due to long-lasting impulsive force causing from high blood flow velocity due to the stenosis.

## 4 Conclusion

A diaphragm in the CCA is very rare. In our case, the young female patient was discovered as an incidental finding and one hypothesis that incomplete regression of the third bronchial arch on aspect of embryology process was proposed. This unusual and previously unreported case is presented to alert vascular sonographers and radiologists to its existence. For such an incidentally and cannot readily be explained aneurismal enlargement with an intact vessel wall structure of carotid artery, sonographers should search the proximal and distal segment of enlargement part carefully to exclude the diaphragm-like structure.
